# The Science of Medicine

**Published:** 1859-03

**Authors:** 


### THE SCIENCE OF MEDICINE.

A philosophical writer in the American Homeopathic Review of New York, utters the grand practical sentiment that "pathology is submitted to a sucession of forms, consistent with the different strata or ages of society." There are two important ideas suggested by this statement, which we will state in language familiar to the masses. Pathology is the science of disease, but the same disease in a day laborer is different from what it is in one whose occupation is sedentary, in a body of health for example, or in one who has a frail constitution. Hence a treatment which would cure a day laborer might kill a weakly woman. Hence the absurdity of using patent medicines, even taking the huge impossibility for granted, that one "certificate" in a thousand is fully true.

Another important inference which very many physicians will strongly object to, because it is an idea ground into them with great assiduity by the professors of medical schools, which possess hospital facilities, is that those facilities are indispensible to the making of an able medical practitioner. But the advantages in this direction are overrated, simply because coarse and rough people and constitutions are met with in hospitals; but to subject those whose whole modes of life and temperament and general systems are entirely different to the same processes of cure and the same doses of medicine, is most extreme folly. The latter would die by the power of the remedies, while the former, if treated as the latter would die for want of remedies. This is an extreme statement, in order to make the contrast more striking.

There is wide complaint of the incompetency of our young doctors, and no stronger proof of this is needed than in the fact that so many soon abandon their profession for other callings; not a few resort to dishonorable means of obtaining practice, whilst multitudes barely succeed in making both ends meet at the end of a lifetime, and even this, not seldom, is attained by painful economies.

Another reason for the incompetence of medical graduates, is their haste to get into practice, and the facility of doing it by means of two courses of lectures, embracing a period of eight months. Unless there is some remedy found for this, we had better go back to the old plan of putting our sons in the office of a village practitioner as an apprentice for two or three years, then gradually taking the master's place in common cases, going round with him from patient to patient, and be thus taught the first great essential of a successful physician, to observe closely and justly, and ask his preceptor all questions freely and fearlessly. Does the young man who walks the wards of the hospital in company with a dozen or two at the heels of his "Professor" do such a thing once a week?—Let them answer.

Another suggestion made by the quotation is this. The same disease in different classes of society, requires a corresponding difference of treatment. But this is only the half of a great truth. The same disease must have a treatment modified by the locality of the patient, by the country, and by the generation or age. And as these are constantly changing, the treatment of the disease that goes by the same name is changed. The man who treats a bilious fever to-day, as it was treated in the last generation, or thirty-three and a third years ago, would kill half of his patients at least. An age ago, bleeding to fainting was considered the great cure all, as indispensible in many forms of severe disease, the man who would follow that practice now, in diseases which bear the same name, would be considered demented. Some of us, not very old either, can muster up reminiscences not particularly delightful, in fact horrible, at the very mention of "salts and senna," or "calomel and jalap," or "cream of tartar and jalap;" and yet, for the same diseases for which these things were considered most potent and indispensible once, not one physician in a thousand now administers them. In fact diseases come and go as do the fashions. Once "every body" had dyspepsia, then clergyman's sore throat was the rage, and now, don't every third person have same form of neuralgia?

These incontrovertible facts lead us to appreciate the depth of truth contained in an editorial of the Medical and Surgical Reporter of Philadelphia. "The profession are too much inclined to follow a routine practice," and hence very properly discourages the publication of books which contain the modes of preparing medicines, with their doses. Such books are worse than useless, they are positively mischievous, they retard progress. The way for any young physician to become successful, is to study out the formulas, the doses of medicine which answer the best purpose in his own locality. We know personally, that one of the most justly eminent surgical and medical practitioners in this country, while at the zenith of his fame, gave the hydriodate of potash in three prescriptions out of four to the drug store, where we had an office for several years. At a period two years later, the hydriodate of potash was seldom mentioned, simply because it had lost its adaptability. This gentleman prescribed from observation and not from books, hence is still a magnate among his brethren. And this brings us to the answer of a question proposed by a southern planter in September last, "Why is it that the medical schools now send such inferior men among us?" The reason is simply this—the student very naturally reveres his professor and preceptor, believes him to be none other than Sir Oracle. The professor states what he gave when he was a young man, and what marvels it performed; the student jumps to the conclusion that what was efficient in his preceptor's youth, must be equally so in his own hands, and he goes out from the green room, with diploma in hand, with the utmost confidence of curing all curable diseases, and of accomplishing like wonders with his preceptor, with a like weapon, when like as not, the very first essay is met with the most signal failure.

If then, the professors in our medical schools really desire to elevate the profession, and will suffer a word of exhortation from one, quite as "regular" as the very foremost of them, being an allopathic dyed in the wool, and from one of the first schools in the Union, after having taken the second collegiate degree, we will give utterance to our wisdom in the words following, to wit—spend more time in teaching young men the principles of medicine, and how to apply them. Teach them to observe what passes before them, rather than to remember what they have read and heard as to theories. Do not lumber up their brains with formulas and endless combinations of quantities and qualities, remembering that a single half dozen of true medical principles thoroughly understood, are of more worth in the making of a skilful practitioner, than all the medicines in the universe; for we are not far from the conviction that medical science as it now is, for the most part is one half figment and the other half fudge, as to its certainties, and will continue so to be, until our medical colleges require, as a condition of graduation, a thorough collegiate education; a thorough knowledge of anatomy and physiology, and of those general principles of health and disease, which are a part of established medicine; and even then, if the mind of the candidate does not accustom itself to a minute attention and careful consideration of the various phenomena presented, he cannot be a good medical scholar nor become a safe or successful physician.

